# A Posterior Incision in Operations for Appendicitis

**Published:** 1904-11-12

**Authors:** 


### A POSTERIOR INCISION IN OPERATIONS FOR APPENDICITIS.

Human ingenuity has not yet been exhausted in the search after novel plans for dealing with the diseased appendix. While every year sees large accessions to the ranks of those who advocate early operation and a corresponding shrinkage in the numbers of those who regard the knife as the last resource and forlorn hope, there has been no such marked difference of opinion as to the site or character of the incision through which the appendix may be reached. Dr. Sheldon,[1] however, comes forward to recommend a new incision altogether; and it must be confessed that he makes out a fairly strong case, on paper, for his method of approaching the appendix from the loin. He has found that when the patient is lying recumbent the appendix is below the level of Petit's triangle; while, if the patient be placed in the latero-prone position, with a large pad under the loin, the cæcum is raised above the iliac crest; so that a diseased appendix or a collection of pus in its neighbourhood can be readily approached through the triangle of Petit.

His method is as follows: a vertical incision is made along the outer border of the latissimus dorsi and extends upwards from the crest of the ilium. This exposes the outer border of the quadratus lumborum and the lumbar fascia and aponeurosis of the transversalis, which extend anteriorly from the outer border of the quadratus muscle. The lumbar fascia and transversalis are then divided by a transverse incision close above the iliac crest.

The advantages which Dr. Sheldon claims for this method over the anterior incision are: (1) in obese subjects it is quicker; (2) it is less likely to be followed by hernia; (3) there are less shock and abdominal distress afterwards; (4) drainage can be more satisfactorily employed; (5) the patients can be allowed up at an earlier date. Dr. Sheldon has operated on 58 cases by this method, and has not yet observed any disadvantage. No large nerves need be injured, and he has never found it necessary to ligate a vessel in performing the operation.

[1] Annals of Surgery, Sept. 1904.

